# Supplementary material for: Contextualizing involvement in terrorist violence by considering non-significant findings: Using null results and temporal perspectives to better understand radicalization outcomes
Source: PLoS One. 2023 Nov 10;18(11):e0292941. doi: 10.1371/journal.pone.0292941 (PMC10637664; doi:10.1371/journal.pone.0292941)
Supplement: S1 Table — (PDF) [file pone.0292941.s001.pdf]

S1 Table. Missing data procedures for all variables

Variable	Valid cases	Missing data	Conviction comparison	Outcome comparison	Gender comparison	Year of birth	Action(s)
Conflict "spillover" influenced radicalization onset	201	2.4%	X^2 (1, N = 206) = 0.21, p = .65	X^2 (1, N = 206) = 5.12, p = .02	X^2 (1, N = 206) = 0.58, p = .45	t = -0.80	***None.*** *Missing at random.*
Presence of a "foreign fighter" locale	205	<1%	X^2 (1, N = 206) = 1.01, p = .32	X^2 (1, N = 206) = 1.01, p = .32	X^2 (1, N = 206) = 0.11, p = .74	t = 0.49	***None.*** *Missing at random.*
(Partial) political representation prior radicalization	56 (out of a possible 176)	68%	X^2 (1, N = 206) = 25.11, p = .00	X^2 (1, N = 206) = 9.81, p = .00	X^2 (1, N = 206) = 0.02, p = .88	t = 5.20***	***Missing not at random.*** *Exclude from prevalence reporting.*
(Partial) political representation during radicalization	181 (70)	12%	X^2 (1, N = 206) = 2.23, p = .14	X^2 (1, N = 206) = 0.05, p = .83	X^2 (1, N = 206) = 1.05, p = .31	t = 1.33	***None.*** *Missing at random.*
Perception of excessive use of force by state(s) prior	174	15.5%	X^2 (1, N = 206) = 14.80, p = .00	X^2 (1, N = 206) = 5.32, p = .02	X^2 (1, N = 206) = 0.03, p = .87	t = 2.74**	***Missing not at random.*** *Exclude from prevalence reporting.*
Perception of excessive use of force by state(s) during	196	4.9%	X^2 (1, N = 206) = 1.68, p = .20	X^2 (1, N = 206) = 0.42, p = .52	X^2 (1, N = 206) = 1.19, p = .28	t = 1.69	***None.*** *Missing at random.*

(Para)military background	200	2.9%	X^2 (1, N = 206) = 0.68, p = .41	X^2 (1, N = 206) = 0.68, p = .41	X^2 (1, N = 206) = 0.70, p = .40	t = 0.57	***None.*** *Missing at random.*
Group members have (para)military expertise	120 (out of a possible 144)	16.7%	X^2 (1, N = 144) = 0.27, p = .60	X^2 (1, N = 144) = 4.52, p = .04	X^2 (1, N = 144) = 4.81, p = .04	t = -0.56	***Missing not at random.*** *Exclude from prevalence reporting as percentage of missing values is > 5% and those with missing data were most likely to be non-involved in terrorist violence.*
Group exerted strong control over behavior / views	128 (out of a possible 144)	11%	X^2 (1, N = 206) = 3.18, p = .08	X^2 (1, N = 206) = 3.82, p = .05	X^2 (1, N = 206) = 0.18, p = .67	t = 0.09	***Missing not at random.*** *Exclude from prevalence reporting as percentage of missing values is > 5% and those with missing data were most likely to be involved in terrorist violence.*
Highest level of education completed prior to radicalization	203	1.5%	X^2 (1, N = 206) = 3.04, p = .08	X^2 (1, N = 206) = 3.04, p = .08	X^2 (1, N = 206) = 0.35, p = .55	t = -0.07	***None.*** *Missing at random.*
Educational enrolment during radicalization	206	0%	***No action. No missing data.***				
Abandonment education during radicalization[a]	128 (out of a possible 129)	<1%	X^2 (1, N = 206) = 2.06, p = .15	X^2 (1, N = 206) = 2.06, p = .15	X^2 (1, N = 206) = 0.86, p = .35	F = 3.08	***None.*** *Missing at random.*
Employed prior to radicalization	204	1%	X^2 (1, N = 206) = 0.00, p = 1.00	X^2 (1, N = 206) = 0.00, p = 1.00	X^2 (1, N = 206) = 0.23, p = .63	t = 0.49	***None.*** *Missing at random.*
Developmental issues (diagnosis)	185	10.2	X^2 (1, N = 206) = 0.05, p = .82	X^2 (1, N = 206) = 0.05, p = .82	X^2 (1, N = 206) = 0.75, p = .38	t = -0.61	***None.*** *Missing at random.*

Employed or in school during radicalization	203<br>Employed (127)<br>In school (33)	1.5%	X^2 (1, N = 206) = 0.00, p = 1.00	X^2 (1, N = 206) = 0.00, p = 1.00	X^2 (1, N = 206) = 0.23, p = 0.63	t = -0.49	***None.*** *Missing at random.*
Mental health difficulties pre-radicalization	189	8%	X^2 (1, N = 206) = 0.58, p = .45	X^2 (1, N = 206) = 3.14, p = .08	X^2 (1, N = 206) = 0.38, p = .54	t = -0.08	See below.
Mental health difficulties during radicalization	198	4%	X^2 (1, N = 206) = 0.52, p = .47	X^2 (1, N = 206) = 8.32, p = .00	X^2 (1, N = 206) = 0.05, p = .83	t = -0.94	**Missing not at random.** Values adjusted.[1]
Substance abuse before radicalization	196	4.9%	X^2 (1, N = 206) = 0.42, p = 0.52	X^2 (1, N = 206) = 1.68, p = 0.20	X^2 (1, N = 206) = 1.19, p = 0.28	t = -1.02	***None.*** *Missing at random*
Substance abuse during radicalization	202	1.9%	X^2 (1, N = 206) = 1.02, p = 0.31	X^2 (1, N = 206) = 0.00, p = 1.00	X^2 (1, N = 206) = 0.46, p = 0.50	t = -1.19	***None.*** *Missing at random*
Negative body-image before radicalization	68	66.9%	X^2 (1, N = 206) = 19.76, p = 0.00	X^2 (1, N = 206) = 5.62, p = 0.02	X^2 (1, N = 206) = 0.90, p = 0.34	t = -0.14	***Missing not at random.*** *Exclude from prevalence reporting.*
Non-violent crime prior	199	3.4%	X^2 (1, N = 206) = 1.33, p = 0.25	X^2 (1, N = 206) = 0.15, p = 0.70	X^2 (1, N = 206) = 0.82, p = 0.36	t = -0.32	***None.*** *Missing at random*
Non-violent crime during	202	1.9%	X^2 (1, N = 206) = 0.00, p = 1.00	X^2 (1, N = 206) = 1.02, p = 0.31	X^2 (1, N = 206) = 0.46, p = 0.50	t = 0.0	***None.*** *Missing at random.*

Violent crime prior	204	1.0%	X^2 (1, *N* = 206) = 0.00, *p* = 1.00	X^2 (1, *N* = 206) = 0.00, *p* = 1.00	X^2 (1, *N* = 206) = 0.23, *p* = 0.63	*t* = -1.23	***None.*** *Missing at random*
Violent crime during	202	1.9%	X^2 (1, *N* = 206) = 1.02, *p* = 0.31	X^2 (1, *N* = 206) = 1.02, *p* = 0.31	X^2 (1, *N* = 206) = 0.98, *p* = 0.32	*t* = 3.40*	***Report cautiously.*** *Potentially missing not at random. When reporting in the context of age, include footnote saying that those with missing data for this variable were significantly older than valid cases.*
Delinquent peers before radicalization	170	17.5%	X^2 (1, *N* = 206) = 4.85, *p* = 0.03	X^2 (1, *N* = 206) = 0.54, *p* = 0.46	X^2 (1, *N* = 206) = 0.17, *p* = 0.69	*t* = -1.27	***Missing not at random.*** *Exclude from prevalence reporting.*
Criminal gang membership before radicalization	203	1.5%	X^2 (1, *N* = 206) = 0.34, *p* = 0.56	X^2 (1, *N* = 206) = 0.34, *p* = 0.56	X^2 (1, *N* = 206) = 0.35, *p* = 0.56	*t* = -3.62***	***Report cautiously.*** *When reporting in the context of age, include footnote saying that those with missing data for this variable were significantly older (average YoB 1954) than valid cases (average YoB 1980).*
Perpetrator of abuse before radicalization	189	8.3%	X^2 (1, *N* = 206) = 0.06, *p* = 0.80	X^2 (1, *N* = 206) = 5.19, *p* = 0.02	X^2 (1, *N* = 206) = 2.10, *p* = 0.15	*t* = -0.32	***None.*** *Missing at random.*
Relationship before	192	6.8%	X^2 (1, *N* = 206) = 2.76, *p* = 0.10	X^2 (1, *N* = 206) = 1.23, *p* = 0.27	X^2 (1, *N* = 206) = 0.71, *p* = 0.19	*t* = 0.32	***None.*** *Missing at random.*
Relationship during radicalization	205	< 1 %	X^2 (1, *N* = 206) = 1.01, *p* = 0.32	X^2 (1, *N* = 206) = 1.01, *p* = 0.32	X^2 (1, *N* = 206) = 0.11, *p* = 0.74	*t* = 1.06	***None.*** *Missing at random.*

Relationship direction during	111 [Of 111]	0%	-	-	-	-	***None*.** *No missing data.*
Children born prior	205	< 1 %	X^2 (1, N = 206) = 1.01, p = 0.32	X^2 (1, N = 206) = 1.01, p = 0.32	X^2 (1, N = 206) = 0.11, p = 0.74	t = 0.18	***None.*** *Missing at random.*
Children born during	205	< 1 %	X^2 (1, N = 206) = 1.01, p = 0.32	X^2 (1, N = 206) = 1.01, p = 0.32	X^2 (1, N = 206) = 0.11, p = 0.74	t = -0.23	***None.*** *Missing at random.*
Living alone before radicalization	191	7.3%	X^2 (1, N = 206) = 1.80, p = 0.18	X^2 (1, N = 206) = 0.07, p = 0.79	X^2 (1, N = 206) = 0.17, p = 0.68	t = -2.04*	***Missing not at random.*** *Those with missing data for this variable were significantly older (average YoB 1973) than valid cases (average YoB 1980).*
Living alone during radicalization	195	5.3%	X^2 (1, N = 206) = 0.86, p = 0.35	X^2 (1, N = 206) = 4.71, p = 0.03	X^2 (1, N = 206) = 0.02, p = 0.91	t = -1.32	***Missing not at random.*** *Exclude from prevalence reporting.*
Homeless before radicalization	205	<1%	X^2 (1, N = 206) = 1.01, p = 0.32	X^2 (1, N = 206) = 1.01, p = 0.32	X^2 (1, N = 206) = 0.11, p = 0.74	t = -2.43*	***Report cautiously.*** *When reporting in the context of age, include footnote saying that the case with missing data for this variable was significantly older (average YoB 1950) than valid cases (average YoB 1979).*
Homeless during radicalization	204	1%	X^2 (1, N = 206) = 0.00, p = 1.00	X^2 (1, N = 206) = 0.00, p = 1.00	X^2 (1, N = 206) = 0.23, p = 0.63	t = 0.19	***None.*** *Missing at random.*
Convert before radicalization	201	2.4%	X^2 (1, N = 206) = 1.85, p = 0.17	X^2 (1, N = 206) = 0.21, p = 0.65	X^2 (1, N = 206) = 0.54, p = 0.46	t = 1.49	***None.*** *Missing at random.*

Jihadist sub-group only	99 of 103	3.9%	-	X^2 (1, N = 103) = 0.09, p = 1.00	X^2 (1, N = 103) = 0.89, p = 0.37	t = 1.67	***None.*** *Missing at random.*
Convert during radicalization	205	<1%	X^2 (1, N = 206) = 1.06, p = 0.32	X^2 (1, N = 206) = 1.01, p = 0.32	X^2 (1, N = 206) = 0.11, p = 0.74	t = 1.63	***None.*** *Missing at random.*
Jihadist sub-group only	102 of 103	1.0%	-	X^2 (1, N = 206) = 0.75, p = 1.00	X^2 (1, N = 206) = 0.12, p = 1.00	t = 1.77	***None.*** *Missing at random.*
Involuntary exit from the military before radicalization	29 of 30	3.3%	X^2 (1, N = 206) = 0.32, p = 1.00	X^2 (1, N = 30) = 0.69, p = 1.00	X^2 (1, N = 30) = 0.04, p = 1.00	t = -0.02	***Report cautiously.*** *Missing not at random but small number of observations due to it being a sub-group analysis.*
Self-esteem before radicalization	104	49.5%	X^2 (1, N = 206) = 11.19, p = 0.00	X^2 (1, N = 206) = 6.29, p = 0.01	X^2 (1, N = 206) = 0.54, p = 0.46	F = .07	***Missing not at random.*** *Exclude from prevalence reporting.*
Attitude towards political system before radicalization	117	43.2%	X^2 (1, N = 206) = 12.36, p = 0.00	X^2 (1, N = 206) = 7.14, p = 0.01	X^2 (1, N = 206) = 0.25, p = 0.62	F = 5.60*	***Missing not at random.*** *Exclude from prevalence reporting.*
Attitude towards political system during radicalization[a]	202	1.9%	X^2 (1, N = 206) = 1.02, p = 0.31	X^2 (1, N = 206) = 1.02, p = 0.31	X^2 (1, N = 206) = 7.06, p = 0.01	F = .36	***Report cautiously.*** *Potentially missing not at random. When reporting in the context of gender, include footnote saying that females were more likely to have missing data for this variable.*
Religious practice before radicalization	113	13.6%	X^2 (1, N = 206) = 0.16, p = 0.33	X^2 (1, N = 206) = 0.00, p = 1.00	X^2 (1, N = 206) = 0.01, p = 0.92	t = 0.29	***None.*** *Missing at random.*

Total time radicalized	203	1.5%	X^2 (1, N = 206) = 0.33, p = 0.68	X^2 (1, N = 206) = 0.34, p = 0.56	X^2 (1, N = 206) = 1.78, p = 0.18	t = -0.30	***None.*** *Missing at random.*
Social isolation prior	202	1.9%	X^2 (1, N = 206) = 1.02, p = 0.31	X^2 (1, N = 206) = 1.02, p = 0.31	X^2 (1, N = 206) = 0.46, p = 0.50	t = -0.54	***None.*** *Missing at random.*
Social isolation during radicalization	205	< 1 %	X^2 (1, N = 206) = 1.00, p = 0.32	X^2 (1, N = 206) = 1.00, p = 0.32	X^2 (1, N = 206) = 0.11, p = 0.74	t = 1.55	***None.*** *Missing at random.*
Family involved in extremism/terrorism	204	1%	X^2 (1, N = 206) = 0.00, p = 1.00	X^2 (1, N = 206) = 2.02, p = 0.16	X^2 (1, N = 206) = 0.23, p = 0.63	t = -0.55	***None.*** *Missing at random.*
Socialized into radical or extreme worldview (childhood)	193	6%	X^2 (1, N = 206) = 0.08, p = 0.77	X^2 (1, N = 206) = 6.65, p = .01	X^2 (1, N = 206) = 0.10, p = .76	t = 0.74	**Missing not at random.** Values adjusted.[2]
Parental divorce	196	4.9%	X^2 (1, N = 206) = 0.42, p = .52	X^2 (1, N = 206) = 0.42, p = 0.52	X^2 (1, N = 206) = 1.19, p = .28	t = -4.02***	***Report cautiously.*** *Potentially missing not at random. When reporting in the context of age include footnote reporting higher proportion of missing values from older subjects.*
Parents involved in crime	164	20.4%	X^2 (1, N = 206) = 0.48, p = .48	X^2 (1, N = 206) = 0.48, p = .48	X^2 (1, N = 206) = 0.54, p = .46	t = -1.80	***None.*** *Missing at random.*

Death of a parent	198	3.9%	X^2 (1, N = 206) = 0.52, p = .47	X^2 (1, N = 206) = 0.52, p = .47	X^2 (1, N = 84) = 0.95, p = .33	t = -3.71***	***Report cautiously.*** *Potentially missing not at random. When reporting in the context of age include footnote reporting higher proportion of missing values from older subjects.*
Family cohesion	164	20.4%	X^2 (1, N = 206) = 7.66, p = .01	X^2 (1, N = 206) = 7.66, p = .01	X^2 (1, N = 84) = 3.52, p = .06	t = -0.37	***Missing not at random.*** *Exclude from prevalence reporting.*
Parental involvement in upbringing / schooling	169	18.0%	X^2 (1, N = 206) = 0.30, p = .59	X^2 (1, N = 206) = 7.41, p = .01	X^2 (1, N = 84) = 2.77 p = .20	t = -3.00**	***Missing not at random.*** *Exclude from prevalence reporting.*
Parental norms	146	29.1%	X^2 (1, N = 206) = 4.61, p = .03	X^2 (1, N = 206) = 0.09, p = .76	X^2 (1, N = 206) = 0.32, p = .57	t = -0.21	***Missing not at random.*** *Exclude from prevalence reporting.*
Number of adults in parental roles during youth	193	6.3%	X^2 (1, N = 206) = 0.08, p = .77	X^2 (1, N = 206) = 0.08, p = .77	X^2 (1, N = 206) = 1.58, p = .21	t = -2.94**	***Missing not at random.*** *Exclude from prevalence reporting.*
Neighborhood socioeconomic status during youth	174	15.5%	X^2 (1, N = 206) = 2.37, p = .12	X^2 (1, N = 206) = 9.47, p = .00	X^2 (1, N = 206) = 1.22, p = .27	t = -0.05	***Missing not at random.*** *Exclude from prevalence reporting.*
Neighborhood socioeconomic status during radicalization	164	20.4%	X^2 (1, N = 206) = 4.31, p = .04	X^2 (1, N = 206) = 0.00, p = 1.00	X^2 (1, N = 206) = 0.03, p = .87	t = -2.84**	***Missing not at random.*** *Exclude from prevalence reporting.*
Number of siblings	198	3.9%	X^2 (1, N = 206) = 0.52, p = .47	X^2 (1, N = 206) = 0.00, p = 1.00	X^2 (1, N = 206) = 0.95, p = .33	t = -0.94	***None.*** *Missing at random.*

SEC parents	142	31%	X^2 (1, N = 206) = 3.91, p = .05	X^2 (1, N = 206) = 0.00, p = 1.00	X^2 (1, N = 84) = .00, p = .95	t = -2.76	***Missing not at random.*** *Exclude from prevalence reporting.*
Mother age at birth of individual	124	39.8%	X^2 (1, N = 206) = 8.10, p = .00	X^2 (1, N = 206) = 0.08, p = 0.78	X^2 (1, N = 206) = 0.41, p = 0.52	t = -1.42	***Missing not at random.*** *Exclude from prevalence reporting.*
Radicalization initiated (partly) through online domain	128	37.9%	X^2 (1, N = 206) = 18.57, p = 0.00	X^2 (1, N = 206) = 0.33, p = 0.57	X^2 (1, N = 206) = 5.53, p = .02	t = -9.09***	***Missing not at random.*** *Exclude from prevalence reporting.*
Viewed explicitly violent imagery before radicalization	137	33.5%	X^2 (1, N = 206) = 7.87, p = 0.01	X^2 (1, N = 206) = 9.61, p = 0.00	X^2 (1, N = 206) = 0.99, p = 0.32	t = 1.37	***Missing not at random.*** *Exclude from prevalence reporting.*
Identifying with victims of perceived injustice before radicalization	173	16.0%	X^2 (1, N = 206) = 4.37, p = 0.04	X^2 (1, N = 206) = 4.37, p = 0.04	X^2 (1, N = 206) = 2.21, p = 0.14	t = 1.45	***Missing not at random.*** *Exclude from prevalence reporting.*
Identifying with victims of perceived injustice during radicalization	186	9.7%	X^2 (1, N = 206) = 0.89, p = 0.35	X^2 (1, N = 206) = 0.89, p = 0.35	X^2 (1, N = 206) = 0.00, p = 0.98	t = 1.32	***None.*** *Missing at random.*
Perceived in-group threat before radicalization	172	16.5%	X^2 (1, N = 206) = 0.14, p = 0.71	X^2 (1, N = 206) = 3.52, p = 0.06	X^2 (1, N = 206) = 0.08, p = 0.77	t = 0.47	***Missing not at random.*** *Exclude from prevalence reporting.*

Perceived existential threat during radicalization	197	4.4%	X^2 (1, N = 206) = 0.12, p = 0.73	X^2 (1, N = 206) = 1.05, p = 0.31	X^2 (1, N = 84) = 1.07, p = .30	t = 1.36	***None.*** *Missing at random.*
Grievance before radicalization	190	7.8%	X^2 (1, N = 206) = 4.34, p = 0.04	X^2 (1, N = 206) = 1.08, p = 0.30	X^2 (1, N = 206) = 0.30, p = 0.59	t = 0.62	***None.*** *Missing at random.*
Grievance during radicalization	203	1.5%	X^2 (1, N = 206) = 0.34, p = 0.56	X^2 (1, N = 206) = 3.04, p = 0.08	X^2 (1, N = 206) = 0.35, p = 0.56	t = 0.03	***None.*** *Missing at random.*
Cognitive opening / unfreezing as radicalization start	187	9.2%	X^2 (1, N = 206) = 4.70, p = 0.03	X^2 (1, N = 206) = 9.80, p = 0.03	X^2 (1, N = 206) = 0.56, p = 0.46	t = 0.92	***Missing not at random.*** *Exclude from prevalence reporting.*
Radicalization driven by clear significance quest	165	19.9%	X^2 (1, N = 206) = 0.03, p = 0.87	X^2 (1, N = 206) = 22.20, p = 0.00	X^2 (1, N = 206) = 3.37, p = 0.07	t = -0.52	***Missing not at random.*** *Exclude from prevalence reporting.*
Extremist role model prior to radicalization	200	2.9%	X^2 (1, N = 206) = 0.00, p = 1.00	X^2 (1, N = 206) = 0.69, p = 0.41	X^2 (1, N = 206) = 3.61, p = 0.06	t = -0.43	***None.*** *Missing at random.*
Recruited into an extremist/terrorist group	122	15.3%	X^2 (1, N = 206) = 5.04, p = .02	X^2 (1, N = 206) = 14.77, p = .00	X^2 (1, N = 206) = 1.31, p = 0.25	t = 0.15	***Missing not at random.*** *Exclude from prevalence reporting.*
Benefits pre	142	31%	X^2 (1, N = 206) = 7.34, p = .01	X^2 (1, N = 206) = 26.20, p = .00	X^2 (1, N = 206) = 1.57, p = .21	t = 1.51	

Group members only	110	23.6	X^2 (1, N = 206) = 9.29, p = .00	X^2 (1, N = 206) = 24.38, p = .00	X^2 (1, N = 206) = 3.36, p = .07	t = 1.43	***Missing not at random.*** *Exclude from prevalence reporting.*
Benefits post	151	27%	X^2 (1, N = 206) = 5.58, p = .02	X^2 (1, N = 206) = 33.96, p = .00	X^2 (1, N = 206) = 1.84, p = .17	t = 1.52	
Group members only	117	18.8%	X^2 (1, N = 206) = 12.60, p = .00	X^2 (1, N = 206) = 27.86, p = .00	X^2 (1, N = 206) = 2.09, p = .15	t = 1.60	***Missing not at random.*** *Exclude from prevalence reporting.*
Role sought or given in group	121	16.0%	X^2 (1, N = 206) = 2.31, p = .13	X^2 (1, N = 206) = 16.42, p = .00	X^2 (1, N = 206) = 0.25, p = .61	t = -0.42	***Missing not at random.*** *Exclude from prevalence reporting.*
Role adoption in group driven by desire for significance	97	32.6%	X^2 (1, N = 206) = 0.60, p = .44	X^2 (1, N = 206) = 19.13, p = .00	X^2 (1, N = 206) = 6.28, p = .01	t = -0.21	***Missing not at random.*** *Exclude from prevalence reporting.*
Potential for material gain influenced radicalization	203	1.5%	X^2 (1, N = 206) = 3.04, p = 0.08	X^2 (1, N = 206) = 0.33, p = 0.56	X^2 (1, N = 206) = 0.35, p = 0.56	t = -2.09*	***Report cautiously.*** *When reporting in the context of age include footnote reporting higher proportion of missing values from older subjects (n = 3).*
Perceived by others as overconfident	174	15.5%	X^2 (1, N = 206) = 0.00, p = 1.00	X^2 (1, N = 206) = 5.33, p = .02	X^2 (1, N = 206) = 0.22, p = .64	t = -1.50	***Missing not at random.*** *Exclude from prevalence reporting.*

Radicalization process driven by desire for revenge	178	13.6%	X^2 (1, N = 206) = 8.10, p = .00	X^2 (1, N = 206) = 20.01, p = .00	X^2 (1, N = 206) = 3.68, p = .06	t = 1.67	***Missing not at random.*** *Exclude from prevalence reporting.*
Relative deprivation before radicalization	157	23.8%	X^2 (1, N = 206) = 0.24, p = 0.08	X^2 (1, N = 206) = 7.74, p = .01	X^2 (1, N = 206) = 2.62, p = .12	t = 0.13	***Missing not at random.*** *Exclude from prevalence reporting.*
Relative deprivation during radicalization	170	17.5%	X^2 (1, N = 206) = 3.37, p = 0.07	X^2 (1, N = 206) = 4.85, p = .03	X^2 (1, N = 206) = 1.03, p = .31	t = 0.05	***Missing not at random.*** *Exclude from prevalence reporting.*
Viewpoint diversity prior	206	0%	-	-	-	-	***None.*** *No missing data.*
Viewpoint diversity during radicalization	206	0%	-	-	-	-	***None.*** *No missing data.*
Viewpoint diversity direction	183	1.6	X^2 (1, N = 186) = 0.28, p = 0.60	X^2 (1, N = 186) = 0.43, p = 0.51	X^2 (1, N = 186) = 0.37, p = 0.54	t = -0.08	***None.*** *No missing data.*
Duty of care before radicalization	206	0.5%	X^2 (1, N = 206) = 1.01, p = 0.32	X^2 (1, N = 206) = 1.01, p = 0.32	X^2 (1, N = 206) = 0.11, p = 0.74	t = 0.18	***None.*** *Missing at random.*
Duty of care during radicalization	206	0.5%	X^2 (1, N = 206) = 1.01, p = 0.32	X^2 (1, N = 206) = 1.01, p = 0.32	X^2 (1, N = 206) = 0.11, p = 0.74	t = 0.23	***None.*** *Missing at random.*
Notable stressors before radicalization	183	11.2%	X^2 (1, N = 206) = 3.96, p = .05	X^2 (1, N = 206) = 2.40, p = 0.12	X^2 (1, N = 206) = 0.64, p = 0.80	t = 0.25	***Missing not at random.*** *Exclude from prevalence reporting.*
Notable stressors during radicalization	192	6.8%	X^2 (1, N = 206) = 4.91, p = .03	X^2 (1, N = 206) = 7.66, p = 0.01	X^2 (1, N = 206) = 0.28, p = 0.60	t = 1.33	***Missing not at random.*** *Exclude from prevalence reporting.*

Note: Variables were removed from reporting if two of the following criteria were met: > 5% missing data and data missing not at random.

[1] When analyzing, ‘Unknowns’ should be counted as ‘no’ for this variable, both before and during radicalization. For the presence or absence of disorder only, again, ‘unknowns’ should be counted as ‘no’ and ‘notable symptoms’ marked as invalid cases.

[2] When analyzing, ‘Unknowns’ should be counted as ‘no’ for this variable.
