# Supplementary material for: Contextualizing involvement in terrorist violence by considering non-significant findings: Using null results and temporal perspectives to better understand radicalization outcomes
Source: PLoS One. 2023 Nov 10;18(11):e0292941. doi: 10.1371/journal.pone.0292941 (PMC10637664; doi:10.1371/journal.pone.0292941)
Supplement: S1 File — (PDF) [file pone.0292941.s002.pdf]

Project: (Non-) involvement in terrorist violence

Outline for semi-structured interviews

Interviewee: [Name]

Interviewer: [Name]

Location: [Address / Online]

Date & duration: [Day / month / year, XX:XX – YY:YY]

Interview recorded: [Yes / no]

File(s): [Filename, length]

PART 1: INTRODUCTION

Introduction & acquaintance

- Outline the project
- Introduce interviewer
- Discuss importance of consent, form there to protect interviewee privacy and safety

Consent

- No substantive questions without prior (written) consent
- Reiterate key aspects consent form (e.g., interviewee is under no obligation of any kind, free to terminate interview whenever so desired, all transcripts/files to be shared with interviewee, full anonymization before results included in publications, etc.)

PART 2: SUBSTANTIVE DISCUSSION

Main topics of discussion

- Topics / questions drawn from codebook
- Specific questions dependent on where other sources have proven insufficient to code the case.
- Maintain conversational style around core themes, rather than specific (structured) interview questions to avoid 'interrogational' style and allow unforeseen explanations to arise.
- Formally confirm category of participant (i.e., involved, or non-involved in terrorist violence).

Topic 1: [SPECIFY]

- **Sub-topic / question: [SPECIFY]**
- **Sub-topic / question 2: [SPECIFY]**
- **Etc.**

Topic 2: [SPECIFY]

- **Sub-topic / question: [SPECIFY]**
- **Sub-topic / question 2: [SPECIFY]**
- **Etc.**

Et cetera

PART 3: CONCLUSION

- Conclude interview;
- Thank interviewee;
- Remind them of measures taken to ensure their privacy/security;
- Encourage them to contact you with any questions they may have;
- Specify when they can expect to be sent abstract / file of recording;
- Ask whether they may be contacted for potential follow-up questions.

PART 4: POTENTIAL FOLLOW-UP QUESTIONS

- List follow-up questions to arise from transcription / analysis.
