# Supplementary material for: Contextualizing involvement in terrorist violence by considering non-significant findings: Using null results and temporal perspectives to better understand radicalization outcomes
Source: PLoS One. 2023 Nov 10;18(11):e0292941. doi: 10.1371/journal.pone.0292941 (PMC10637664; doi:10.1371/journal.pone.0292941)
Supplement: S2 File — (PDF) [file pone.0292941.s003.pdf]

Interview consent form for the project: '(Non-)involvement in Terrorist Violence'

I agree to cooperate with this research project under the following conditions:

- The project's goal is gain clearer insights into why the majority of people that embrace radical or extremist ideas do not become involved in terrorist violence;

- My cooperation is entirely voluntary. I can decide at any moment to not answer questions or to withdraw my cooperation without this having any negative consequences for myself;

- Information that I provide will not be used to initiate criminal proceedings against myself or others. The only exception to this rule concerns information that points to a direct threat to the physical wellbeing of myself or others;

- The interview will only be recorded if I agree to it;

- All information stemming from my cooperation will be treated as strictly confidential to safeguard the privacy and safety of myself and others;

- All information stemming from my cooperation (e.g. notes, audio recordings) will be safely stored using encryption software;

- Information stemming from my cooperation can only be made public (e.g. in articles, presentations or reports) after it has been thoroughly anonymized. Under no circumstances will details be published that can be used to personally identify myself or others. To do so, all personally-identifying information such as names, dates of birth, place of residence, et cetera will be removed prior to publication;

- Information stemming from my cooperation is only intended for use within this specific project. This information cannot be accessed or used by anyone but Bart Schuurman, nor can it be used for different research projects, without my consent;

- A copy of all notes and/or audio recordings stemming from my cooperation with this project will be sent to me by Bart Schuurman if requested;

- For more information about the project, I can contact dr. Bart Schuurman, Associate Professor at Leiden University. E-mail: b.w.schuurman@fgga.leidenuniv.nl, phone +31 70 800 9347.

Name interviewee	Name interviewer:
Date:	Date:
Signature interviewee:	Signature interviewer:
