# Supplementary material for: Contextualizing involvement in terrorist violence by considering non-significant findings: Using null results and temporal perspectives to better understand radicalization outcomes
Source: PLoS One. 2023 Nov 10;18(11):e0292941. doi: 10.1371/journal.pone.0292941 (PMC10637664; doi:10.1371/journal.pone.0292941)
Supplement: S3 File — (PDF) [file pone.0292941.s004.pdf]

Understanding non-involvement in terrorism: CODEBOOK

Bart Schuurman
Version: 14 December 2021

Background

Introduction

This codebook is part of the project ‘Understanding non-involvement in terrorism’. The overarching premise is that research on involvement in terrorist violence has focused almost exclusively on the small group of people who actually (help) plan or perpetrate terrorist attacks, without considering why the vast majority of those who radicalize never cross the threshold to actual violence. By essentially selecting on the dependent variable, existing studies have failed to consider why most people who radicalize *do not* become involved in terrorist violence.

Goal

This project compares radicalized individuals *not* involved in terrorist violence with radicalized individuals who *are* involved in terrorist violence. This comparison specifically utilizes both risk and protective factors, drawing them from a range of disciplines including terrorism studies, criminology and war studies. All variables are divided into structural, group and individual-level clusters for greater clarity.

This codebook provides a way of collecting detailed biographical information on individuals undergoing a radicalization process, that is structured to allow comparisons and specifically attuned to both risk and protective factors. Once data collection is complete, straightforward descriptive statistics will identify the most salient overall differences between the ‘radicalized/non-involved in terrorist violence’ and the ‘radicalized/involved in terrorist violence’ subsets. It should be noted that the ‘non-involved in terrorist violence’ category can include people engaged in a range of other terrorism-related activities, such as propaganda, recruitment and financing.

Codebook as an iterative product

Given the large number of variables for which data is to be collected and the difficulty of finding secondary and primary sources of sufficient detail and reliability to provide full biographies of the radicalized individuals studied here, there will be numerous instances of variables or cases for which data is incomplete. To minimize the chances of this occurring, the variables selected for inclusion have gone through an initial screening process that removed those that are clearly not applicable to the Western European context from which cases are drawn (e.g. absolute poverty, war) or for which it was clear that data could not be collected (e.g. on the biological determinants of delinquent behavior). As the first cases are coded, the number of remaining variables is likely to decrease further by removal of those for which no or too little data could be found. The final version of the codebook will thus be the product of an iterative process.

Inclusion criteria

This project takes individuals as its unit of analysis, specifically those who are undergoing or have undergone a radicalization process. A distinction is made between those who are 'radicalized / non-involved in terrorist violence' and those who are 'radicalized and involved in terrorist violence'. In terms of ideological orientation, the project is specifically focused on Salafi-Jihadists and right-wing extremists.

Key terms

1. Radicalization – the process of increasing adherence to either radical or extremist ideological / religious convictions. Radical meaning here convictions that advocate far-reaching social/economic/political change but not the complete destruction of the existing order and *usually* without recourse to violent means. Extremist meaning here convictions that advocate far-reaching social/economic/political change that specifically do necessitate the revolutionary overthrow/destruction of the existing order and that emphatically embrace violent means as the only effective and legitimate means of achieving these goals.

2. Involved in terrorist violence – individuals are considered involved in terrorist violence when they engage in behavior that directly leads to the planning, preparation or commission of 'calculated, demonstrative, direct violent action without legal or moral restraints, targeting mainly civilians and non-combatants, performed for its propagandistic and psychological effects on various audiences and conflict parties'.[1]

   Individuals qualified as non-involved in *terrorist violence* may still engage in a range of *terrorism-related* activities such as fund-raising, distribution of propaganda, or recruitment.[2] Individuals who travel to Syria or attempted to do so are currently **not included** as this is seen as a qualitatively different form of political violence than engaging in the planning, preparation or commission of acts of terrorist violence.

Sources

- Open sources where possible (academic publications, media reports, (auto)biographies, sentencing information etc.);
- Privileged information where open sources fall short (interviews, police files).

[1] Schmid, "The Definition of Terrorism," 86–87.

[2] This follows the distinction between violent and non-violent extremists made by Knight, Woodward, and Lancaster, "Violent versus Nonviolent Actors," 236.

Codebook

Subject:
Conviction:
Category
Coder:
Coding start:
Coding end:

CategoryJustification: for those classified as 'involved in terrorist violence', what is the basis?
[ ] Intent to use terrorist violence
[ ] Planning / preparation
[ ] Access to weapons
[ ] Attack executed

STRUCTURAL-LEVEL VARIABLES

1. **SpilloverInvolvement:** Was there an armed conflict ongoing that influenced the desire to explore or adopt an extremist ideology?
   [ ] No
   [ ] Yes
   [ ] Unknown

2. **SpilloverEvent:** Was there an armed conflict ongoing that influenced the desire to act violently on behalf of an extremist ideology?
   [ ] No
   [ ] Yes
   [ ] Unknown
   [ ] Does not apply

3. **FF Locale:** Did the extremist movement at the time offer broadly-recognized opportunities to travel abroad and join insurgent or terrorist groups as a foreign fighter?
   [ ] No
   [ ] Yes
   [ ] Unknown

4. **PoliticalRep:** Did political parties represent (some of) the views the individual adhered to prior to their radicalization? Use DNA only when there is evidence of an absence of interest in politics / absence of a political consciousness.
   [ ] No
   [ ] Yes

[ ] Unknown
[ ] Does not apply

5. **PR_During:** Did political parties represent (some of) the views the individual adhered to during radicalization/involvement?
   [ ] No
   [ ] Yes
   [ ] Unknown
   [ ] Does not apply

6. **PR_DurDirect:** If political parties represented (some of) the views that the individual adhered to during radicalization/involvement, how is this political representation best described throughout the radicalization/involvement period?
   [ ] Degree of representation declines (weakened identification individual-party)
   [ ] Stability
   [ ] Degree of representation increases (stronger identification individual-party)
   [ ] No longer represents views
   [ ] Unknown
   [ ] Does not apply

7. **ExcessiveForcePrior:** Did the individual perceive state forces using excessive force against the group or broader movement with which the individual identified, at home or abroad, prior to radicalization?
   [ ] No
   [ ] Yes
   [ ] Unknown

8. **ExcessiveForceDuring:** Did the individual perceive state forces using excessive force against the group or broader movement with which the individual identified, at home or abroad, during involvement in an extremist group or the broader extremist movement?
   [ ] No
   [ ] Yes
   [ ] Unknown

GROUP / MOVEMENT-LEVEL VARIABLES

9. **ExtremistRoleModel:** Did the group or wider movement (or in some cases ideologically different but equally extreme movements) include individuals who through their behavior or arguments became role models / authority figures who contributed to the subject's adoption of

an extremist ideology / worldview? (Face-to-face = present in social ecology, at a distance = through media, books, etc.)

[ ] No
[ ] Yes, face-to-face
[ ] Yes, at a distance
[ ] Yes both
[ ] Unknown
[ ] Does not apply

10. **ViolentRoleModel_evt:** Did the group or wider movement (or in some cases ideologically different but equally extreme movements) include individuals who through their behavior or arguments became role models / authority figures who contributed to the subject's motivation to carry out an act of terrorist violence?

[ ] No
[ ] Yes, face-to-face
[ ] Yes, at a distance
[ ] Yes both
[ ] Unknown
[ ] Does not apply

11. **Group age:** How long had the group been in existence at the beginning of the subject's involvement process? **Note:** (co-)founders of groups use the time stamp 0,01. Calculate average age if there are multiple or subsequent group memberships.

[ ] *Age in months*
[ ] Unknown
[ ] Does not apply

12. **Group size:** What is the (average) group size in terms of number of participants?

[ ] *Estimated number of members*
[ ] Unknown
[ ] Does not apply

13. **Mil members:** Did group include members with a (para)military background or experience? Only count those who were available to the group; i.e. members who traveled to and stayed in foreign conflict zones do not count as their (para)military experience was not a resource available to the group.

[ ] No
[ ] Yes
[ ] Unknown
[ ] Does not apply

14. **CallsTPV:** Has the group *or movement* advocated in favor of political violence / terrorism (videos claiming responsibility for attacks excluded)? **Please note**: extremist movements are unlikely to have a single view / position on the use of violence. The point of reference here is *the individual's primary point of reference* within the movement. Ie: within the part(s) of the movement that the individual was most inspired by, were there calls for terrorism or political violence?
    - [ ] No
    - [ ] Yes
    - [ ] Unknown
    - [ ] Does not apply

15. **OpInPris:** Does the group *or movement* operate in prison(s)?
    - [ ] No
    - [ ] Yes
    - [ ] Unknown
    - [ ] Does not apply

16. **Publish:** Does the group (so here not the movement!) publish ideological materials (including online)?
    - [ ] No
    - [ ] Yes
    - [ ] Unknown
    - [ ] Does not apply

17. **MoralLogicGroup:** How can the group (*or movement*)'s moral logic towards violence best be described? Similar to #14, take that part of the group/movement to which the individual in question was primarily drawn as a reference point.
    - [ ] Necessary & legitimate, few discussions on boundaries
    - [ ] Qualified (e.g. when, who, how, against whom)
    - [ ] Inappropriate (e.g. never or last resort defensive only)
    - [ ] Not or hardly discussed
    - [ ] Unknown
    - [ ] Does not apply

18. **StratLogicGroup:** How is the group (*or movement*)'s strategic logic best described? Similar to #14, take that part of the group/movement to which the individual in question was primarily drawn as a reference point.
    - [ ] Violence is seen as most effective means
    - [ ] Mixed strategy: violent and non-violent means
    - [ ] Non-violence is seen as most effective means
    - [ ] Not or hardly discussed

[ ] Unknown
[ ] Does not apply

19. **PP_BehaviorViews:** Did the group exert strong control over behavior and / or worldview?
    [ ] No
    [ ] Yes, behavior
    [ ] Yes, worldview
    [ ] Yes, both
    [ ] Unknown
    [ ] Does not apply

20. **CompetitionGroup:** Is there ideological or organizational (e.g. for strategic direction, roles etc.) competition within the group during the subject's participation?
    [ ] No
    [ ] Yes, ideological
    [ ] Yes, organizational
    [ ] Yes, both
    [ ] Unknown
    [ ] Does not apply

21. **CompetitionMovement:** Is there tangible ideological or organizational (e.g. for strategic direction, roles etc.) competition within the broader movement during the subject's participation?
    [ ] No
    [ ] Yes, ideological
    [ ] Yes, organizational
    [ ] Yes, both
    [ ] Unknown
    [ ] Does not apply

22. **SuccessViolentActorsPrior:** Had violent actors achieved noteworthy success (e.g. in terms of media attention or achievement political goals) through the use of violence within the broader ideological movement prior to the subject's involvement as noted by the individual?
    [ ] No
    [ ] Yes
    [ ] Unknown
    [ ] Does not apply

23. **SuccessViolentActorsDuring:** Had violent actors achieved noteworthy success (e.g. in terms of media attention or achievement political goals) through the use of violence within the broader ideological movement during the subject's involvement, as noted by the individual?

[ ] No
[ ] Yes
[ ] Unknown
[ ] Does not apply

INDIVIDUAL-LEVEL VARIABLES

---------------------------------INDIVIDUAL BACKGROUND--

24. **YOB:** Year of birth
    [ ] Number

25. **Sex**
    [ ] Male
    [ ] Female

26. **EduObt:** Highest level of education completed prior to radicalization?
    [ ] No school
    [ ] Primary school
    [ ] Secondary school
    [ ] Trade / vocational
    [ ] Bachelor
    [ ] Master
    [ ] PhD
    [ ] Unknown

27. **EduNow:** Enrolled in education during involvement? If yes, indicate highest level.
    [ ] No
    [ ] Yes
    [ ] Unknown

28. **EduDirection:** How can the subject's educational development during involvement best be described?
    [ ] Less time spent on education
    [ ] Completed / stable
    [ ] More time spent on education
    [ ] Abandoned education
    [ ] Unknown
    [ ] Does not apply

29. **EmployedPrior:** Employed before radicalization? Side-jobs during school do not count.
    [ ] No

[ ] No, in school
[ ] Yes
[ ] Unknown

30. **EmployedDuring:** Employed (at some point / for periods) during radicalization / involvement? Side-jobs during school do not count towards employment.
    [ ] No
    [ ] No, in school
    [ ] Yes
    [ ] Unknown

31. **EmploymentDirection:** How can the subject's employment development during involvement best be described?
    [ ] Less time spent on work
    [ ] Stability
    [ ] More time spent on work
    [ ] Abandoned work
    [ ] Unknown
    [ ] Does not apply

32. **Immigrant:** Immigration background or status?[3]
    [ ] No
    [ ] Yes, 1st gen
    [ ] Yes, 2nd gen
    [ ] Yes, 3d gen
    [ ] Unknown

33. **DevelopmIssues:** Did the subject show signs of developmental issues? As per 29 June 2021, this item has been disaggregated into 2 parts:
    - **Diagnosis** (e.g. autism spectrum disorder (ASD), intellectual disability, cerebral palsy, Tourette's syndrome, hearing impairment, vision impairment, speech impairment, epilepsy/seizure disorder, traumatic brain injury/concussion, or other developmental delays). See link for overview.[4]
    - To capture the experiences of individuals who, for whatever reason, did not receive a diagnosis but nonetheless displayed notable indicators of delay, there is an option for cases to display **indicators** of developmental issues. Here, delay is indicative of not

[3] Centraal Bureau voor de Statistiek, "Wat Is Het Verschil."
[4] Centers for Disease Control and Prevention, "Child Development."; see also Udin et al, 2021, p. 183

reaching a certain milestone by 21. Classic examples would include not speaking, walking, progressing through school levels/grades etc. relative to their peers[5].

[ ] No
[ ] Yes, diagnosis
[ ] Yes, indicators
[ ] Unknown

34. **HistMental:** Does the individual have a history of mental-health issues prior to involvement? Per 8 June 2021, this item has been disaggregated into 2 parts:

- Mental **disorder** as specified in DSM: only applicable if there is concrete proof of a diagnosis made by a qualified expert (e.g. psychiatrist, psychologist).
- If there was evidence of *symptoms* of a mental disorder (i.e. insight into the difficulties any individual may have experienced) but, for whatever reason, an individual did not receive a diagnosis (either because a professional could not infer diagnostic levels or because the individual did not have access to services), they were marked as having **notable symptoms**. This category would apply if a professional noted symptoms but didn't feel they had enough information to diagnose (e.g. court appointed professional with limited access to early childhood records or family members). This category is also intended to capture cases' subjective experiences, whilst not saturating the disorder item.

[ ] No
[ ] Yes, disorder(s)
[ ] Yes, notable symptom(s)
[ ] Unknown

35. **CurrentMental:** Did the individual suffer from mental health issues during involvement?

[ ] No
[ ] Yes, disorder(s)
[ ] Yes, notable symptom(s)
[ ] Unknown

36. **HistSubAbuse:** Does the individual have a history of substance abuse / addiction prior to involvement? Determining 'abuse' depends on ascertaining whether the substance use led to

[5] It is important to note that this item does not capture the nuance of delay due to, for example, environmental factors. Evidence suggests that family resilience, family meals, and mother's mental health promote flourishing and school engagement in children with developmental delay (Udin et al, 2021) and the opposite is the case for children emerging from adverse environments (Marie-Mitchell et al., 2013). However, it is beyond the scope and expertise of the current study to determine if a developmental delay would have emerged, had the case been exposed to a less adverse home life (and vice versa).

problems with school, work, family, relationships and/or health. **Please note**: addiction to gambling or gaming can also be captured here.

[ ] No
[ ] Yes
[ ] Unknown

37. **CurrSubAbuse:** Was the individual a substance abuser / addict during involvement?

[ ] No
[ ] Yes
[ ] Unknown

38. **BodyImage:** Has the individual experienced body-image related issues prior to involvement?

[ ] No
[ ] Yes
[ ] Unknown

39. **ContactAuth:** Does the individual have a history of run-ins with the police / authorities short of convictions prior to involvement?

[ ] No
[ ] Yes
[ ] Unknown

40. **CrimeNonViolPrior:** History of non-violent crime prior to involvement?

[ ] No
[ ] Yes
[ ] Unknown

41. **CrimeNV_Dur:** Non-violent crime unrelated to terrorism during involvement?

[ ] No
[ ] Yes
[ ] Unknown

42. **ViolentCrimePrior:** History of violent crime prior to involvement? **Note:** street fights, brawls, where opponents were not unsuspecting but more-or-less willing participants consenting to engage in (unarmed) violence are excluded here (but captured under abuse).

[ ] No
[ ] Yes
[ ] Unknown

43. **ViolentCrimeDur:** Violent crime other than terrorist violence during involvement? **Note:** street fights, brawls, where opponents were not unsuspecting but more-or-less willing

participants consenting to engage in (unarmed) violence are excluded here (but captured under abuse).

[ ] No
[ ] Yes
[ ] Unknown

44. **Prison:** Spent time in prison prior to involvement?

[ ] No
[ ] Yes
[ ] Unknown

45. **PrisonDuring:** Spent time in prison during involvement but prior to final / current arrest or exit?

[ ] No
[ ] Yes
[ ] Unknown

46. **DelinqPeers:** In contact with peers / friends involved in delinquent behavior prior to radicalization / involvement in extremism?

[ ] No
[ ] Yes
[ ] Unknown

47. **GangHist:** History of criminal gang involvement prior to radicalization?

[ ] No
[ ] Yes
[ ] Unknown

48. **VictimAbuse:** Exposure to violence or abuse as victim prior to radicalization?

[ ] No
[ ] Yes
[ ] Unknown

49. **PerpAbuse:** Exposure to violence or abuse as perpetrator prior to radicalization?

[ ] No
[ ] Yes
[ ] Unknown

50. **RelationshipBefore:** In a relationship prior to involvement?

[ ] No

[ ] Yes
[ ] Unknown

51. **RelationshipDuring:** In a relationship during involvement?
    [ ] No
    [ ] Yes
    [ ] Unknown

52. **RelDirection:** If in a relationship, how did that develop during radicalization / involvement?
    [ ] Break-up / divorce
    [ ] Break-up / divorce followed by new rel.
    [ ] Stability
    [ ] Unknown
    [ ] Does not apply

53. **SO_Radicalized:** Significant other involved in radicalism / extremism?
    [ ] No
    [ ] Yes
    [ ] Unknown
    [ ] Does not apply

54. **ChildrenBefore:** Children born prior to involvement?
    [ ] No
    [ ] Yes
    [ ] Unknown

55. **ChildrenDuring:** Children born during involvement?
    [ ] No
    [ ] Yes
    [ ] Unknown

56. **LivesAloneBefore:** Lives alone prior to involvement? **Please note:** scoring this item, consider that it is more relative than absolute. I.e., a ‘yes’ is only appropriate where the subject’s time living alone prior to involvement represents a significant percentage of the pre-involvement timeframe.
    [ ] No
    [ ] Yes
    [ ] Unknown

57. **LivesAloneDuring:** Lives alone during involvement? **Please note:** scoring this item, consider that it is more relative than absolute. I.e., a ‘yes’ is only appropriate where the subject’s time

living alone during involvement represents a significant percentage of the involvement timeframe.
[ ] No
[ ] Yes
[ ] Unknown

58. **HomelessBefore:** Experienced homelessness prior to involvement? Homelessness is defined here as the individual lacking a fixed, regular and adequate nighttime residence.
[ ] No
[ ] Yes
[ ] Unknown

59. **HomelessDuring:** Experienced homelessness during involvement? Homelessness is defined here as the individual lacking a fixed, regular and adequate nighttime residence.
[ ] No
[ ] Yes
[ ] Unknown

60. **ConvertBefore:** Convert or suddenly reawakened religious / ideological interest prior to involvement?
[ ] No
[ ] Yes, convert
[ ] Yes, reawakened
[ ] Unknown

61. **ConvertDuring:** Convert or suddenly reawakened religious / ideological interest during involvement?
[ ] No
[ ] Yes, convert
[ ] Yes, reawakened
[ ] Unknown

62. **SelfControl[6]:** Using the scale below, subject's ability to exert self-control is best defined as:

Concrete here-and-now orientation	
Lack of diligence, tenacity or persistence in a course of action	
Adventuresome, active, and physical	
Indifferent, or insensitive to the suffering and needs of others	
Minimal tolerance for frustration (volatile temper)	

[6] Gibson, "Measurement of Self-Control," 2999.

Little ability to respond to conflict through verbal rather than physical means	

NOTE: Enter score as 6-[number scored with table]

[6-X =]
[ ] Unknown

63. **CogClosure:** did the individual display a clear preference for high cognitive closure?
[ ] No
[ ] Yes
[ ] Unknown

64. **SocSkills:** Individual's social skills as perceived by others are best described as *trending towards*:
[ ] Well-developed (extraverted, communicates well, enjoys company others, etc.)
[ ] Neutral
[ ] Under developed (introverted, communicates poorly, favors being alone, etc.)
[ ] Unknown

65. **SelfEsteem:** Individual's self-esteem prior to involvement is best described as:
[ ] High
[ ] Neutral
[ ] Low
[ ] Unknown

66. **AttitudeSystemPre:** Attitude towards political system / institutions prior to involvement can be best described as:
[ ] Positive
[ ] Neutral
[ ] Negative
[ ] Unknown

67. **AttitudeSystemPost:** Attitude towards political system / institutions post involvement can be best described as:
[ ] Positive
[ ] Neutral
[ ] Negative
[ ] Unknown

68. **HeadInjury:** Suffered serious head injury during childhood?

[ ] No
[ ] Yes
[ ] Unknown

69. **InvolExitMil:** Involuntary exit from military service prior or during involvement?
[ ] No
[ ] Yes, prior
[ ] Yes, during
[ ] Unknown
[ ] Does not apply

70. **MilServNeg:** Perceives military service as being negatively evaluated by others prior to involvement?
[ ] No
[ ] Yes
[ ] Unknown
[ ] Does not apply

71. **(Para)MilExp:** (Para)military experience or training? Note: shooting practice is included under paramilitary.
[ ] No
[ ] Yes, paramilitary
[ ] Yes, military
[ ] Yes, both
[ ] Unknown

72. **AccessWeap:** Access to firearms and/or explosives?
[ ] No
[ ] Yes, firearms
[ ] Yes, explosives
[ ] Yes, both
[ ] Unknown

---------------------------------FAMILY HISTORY---

73. **Sibling#:** Total number of children in family (including subject)
[ ] # of siblings
[ ] Unknown
[ ] Does not apply

74. **FamExtrTerr:** Family members involved in extremism / terrorism prior to involvement?

[ ] No
[ ] Yes
[ ] Unknown
[ ] Does not apply

75. **SocializedRad:** Socialized into radical or extremist worldviews during childhood (up to and including 13 years of age) by family or peers?
[ ] No
[ ] Yes, radical
[ ] Yes, extremist
[ ] Unknown

76. **FamCohesion:** Family cohesion growing up can be typified as *trending towards*:
[ ] Positive (cohesive, warm, absence of discord)
[ ] Neutral
[ ] Negative (fractured, cold, frequent discord)
[ ] Unknown
[ ] Does not apply

77. **ParentSocEcon:** Socio-economic status parents can be typified as:

	Max high school (0)	Trade or BA (1)	MA or more (2)
Education parents **(total for both parents)**			
	Both unemployed (0)	One works (1)	Both work (2)
Employment			
	Below median (0)	Ca. median (1)	Above median (2)
Joint income estimate			
	Bottom 33% (0)	Middle 33% (1)	Top 33% (2)
Employment prestige[7] **(highest parent only)**			

Unknowns are not counted

[ ] Number
[ ] Unknown

78. **ParentInvol:** Parental involvement in upbringing and schooling can be typified as *trending towards*:
[ ] Active
[ ] Neutral

[7] Cörvers et al., "Status En Imago," 69–72.

[ ] Absent
[ ] Unknown
[ ] Does not apply

79. **ParentNorms:** Social norms and values promoted by parents can be typified as *trending towards*:
    [ ] Pro-social
    [ ] Neutral
    [ ] Anti-social
    [ ] Unknown
    [ ] Does not apply

Prosocial seen here as behavior or attitudes that "benefit[s] other people or society as a whole", "such as helping, sharing, donating, co-operating, and volunteering".

Antisocial seen here as behaviors or attitudes that that harm or lack consideration for the well-being of others, violates the basic rights of another person, or is considered to be disruptive to others in society.

80. **Divorced:** Parents divorced or separated?
    [ ] No
    [ ] Yes
    [ ] Unknown

81. **MotherAge:** Age of mother at birth of subject?
    [ ] Number
    [ ] Unknown

82. **ParentRoles:** The number of adults occupying parental roles during upbringing (< 21) was typified by:
    [ ] Frequent change (2 or more changes in father/mother figure)
    [ ] Change (1 change in father / mother figure)
    [ ] Consistency
    [ ] Unknown
    [ ] Does not apply

83. **ParentCrime:** Did parent(s) have conflicts with the law while subject was growing up?
    [ ] No
    [ ] Yes
    [ ] Unknown

[ ] Does not apply

84. **ParentSubAb:** Did parent(s) have a substance abuse problem while subject was growing up?
    [ ] No
    [ ] Yes
    [ ] Unknown
    [ ] Does not apply

85. **Disruptive:** Did the individual experience disruptive life events prior to radicalization / involvement start? Emphasis placed on *subjective experience* of event by subject in determining if it was disruptive or not. But for some general guidance: **regular events** (pregnancy, birth, illnesses, schooling, retirement etc.); **irregular events** (changes in residence, job, core relationships); **disruptive events** (burglaries, assaults, contacts with police or courts, divorce, suicide by relatives, serious quarrels, etc.).[8] Focus here is on irregular and/or disruptive categories.
    [ ] No
    [ ] Yes
    [ ] Unknown
    [ ] Does not apply

86. **DeathParent:** Experienced death of parent while growing up (<21 years of age)?
    [ ] No
    [ ] Yes
    [ ] Unknown
    [ ] Does not apply

87. **NeighbSEHist:** Neighborhood socio-economic status growing up
    [ ] High
    [ ] Middle
    [ ] Low
    [ ] Unknown

88. **NeighbSENow:** Neighborhood socio-economic status at time of radicalization process start.
    [ ] High
    [ ] Middle
    [ ] Low
    [ ] Unknown

--------------------------------INVOLVEMENT DYNAMICS--

[8] Brown and Harris, “Interlude,” 368.

89. **AgeInvolved:** At what (approximate) age did the individual begin radicalization / become involved?
   [ ] Age
   [ ] Unknown

90. **OnlineExtr:** Did access to information and/or contact with radicalized individuals via the Internet play a role in initiating the radicalization process?
   [ ] No
   [ ] Yes
   [ ] Unknown
   [ ] Does not apply

91. **OnlineExtrEvt:** Did access to information and/or contact with radicalized individuals via the Internet contribute to the event decision?
   [ ] No
   [ ] Yes
   [ ] Unknown
   [ ] Does not apply

92. **ViolentImageryBefore:** Watched extremely violent (execution) video's / images before radicalization process start?
   [ ] No
   [ ] Yes
   [ ] Unknown
   [ ] Does not apply

93. **VI_During:** Watched extremely violent (execution) video's / images during radicalization?
   [ ] No
   [ ] Yes
   [ ] Unknown
   [ ] Does not apply

94. **Ideology:** Ideological conviction during involvement can be best typified as:
   [ ] Radical
   [ ] Extremist
   [ ] Mainstream
   [ ] Unknown

95. **IdeoCommit:**[9] Degree of ideological commitment is represented as a three-point scale. Score and tally items below to give a number between 0 (low ideological commitment) and 3 (high ideological commitment).
    [ ] Score
    [ ] Abandoned important responsibilities to focus on convictions / movement
    [ ] Abandoned important relationships to focus on convictions / movement
    [ ] Dedication to ideological tenets of <u>extremist</u> worldview in more than simply appearance / as thin justification for antisocial behavior
    [ ] Unknown

96. **IdentVictimsBefore:** Personal identification with victims of perceived injustice prior to radicalization process start?
    [ ] No
    [ ] Yes
    [ ] Unknown

97. **IdentVictimsDuring:** Personal identification with victims of perceived injustice during radicalization / involvement?
    [ ] No
    [ ] Yes
    [ ] Unknown

98. **InGroupThreatBefore:** Perceives survival own group or community as seriously threatened before radicalization process start?
    [ ] No
    [ ] Yes
    [ ] Unknown
    [ ] Does not apply

99. **InGroupThreatDuring:** Perceives survival own group or community as seriously threatened during radicalization/involvement?
    [ ] No
    [ ] Yes
    [ ] Unknown
    [ ] Does not apply

---

[9] Based in part on Kruglanski, Webber, and Koehler, *The Radical's Journey*.

100. **GrievanceBefore:** Clear grievance against other group(s) / individual(s) prior to radicalization process start?

[ ] No
[ ] Yes, political
[ ] Yes, religious
[ ] Yes, personal
[ ] Yes, political & religious
[ ] Yes, religious & personal
[ ] Yes, political & personal
[ ] Yes, political, religious & personal
[ ] Unknown

101. **GrievanceDuring:** Clear grievance against other group(s) / individual(s) during involvement / radicalization?

[ ] No
[ ] Yes, political
[ ] Yes, religious
[ ] Yes, personal
[ ] Yes, political & religious
[ ] Yes, religious & personal
[ ] Yes, political & personal
[ ] Yes, political, religious & personal
[ ] Unknown

102. **PersOblig:** Felt a personal obligation to act using violence against perceived injustices during involvement / radicalization?

[ ] No
[ ] Yes
[ ] Unknown

103. **FailedForeignFighter**: did the individual try but fail to join a foreign insurgent and/or terrorist organization as a foreign fighter? Failure can be due to a variety of factors, e.g. passport being confiscated by authorities, being stopped at the border, envisioned group not accepting foreign applicants, etc.

[ ] No
[ ] Yes
[ ] Unknown

104. **IntentViolBefore:** Stated intent to commit deadly or terrorist violence before radicalization process start?

[ ] No
[ ] Yes
[ ] Unknown

105. **IV_During:** Stated intent to commit deadly or terrorist violence during involvement / radicalization?
[ ] No
[ ] Yes
[ ] Unknown

106. **ParticipActRadBefore:** Participates in non-violent ideologically-informed activities before radicalization process start (e.g. marches, demonstrations, leafletting)? Activism takes place in the open and is centered on campaigning for change. **Please note:** take care to distinguish between extremist convictions and actual behavior. Participating in demonstrations or electoral politics does not necessarily make the goals pursued any less extremist. Likewise, participation in non-violent activism does not rule out also or later engaging in terrorist violence.
[ ] No
[ ] Yes
[ ] Unknown

107. **ParticipActRadDuring:** Participates in non-violent ideologically-informed activities during involvement / radicalization (e.g. marches, demonstrations, leafletting)? Activism takes place in the open and is centered on campaigning for change. **Please note:** take care to distinguish between extremist convictions and actual behavior. Participating in demonstrations or electoral politics does not necessarily make the goals pursued any less extremist. Likewise, participation in non-violent activism does not rule out also or later engaging in terrorist violence.
[ ] No
[ ] Yes
[ ] Unknown

108. **ActRadDirection:** How can the subject's participation in activism during involvement best be described?
[ ] Less time spent on non-violent activism
[ ] Stability
[ ] More time spent on non-violent activism
[ ] Abandoned non-violent activism
[ ] Unknown
[ ] Does not apply

109. **ParticExtr:** Became involved in extremist group (i.e. one committed to revolutionary change and/or in favor of the use of violence to bring about change)? **Please note for lone actors: this should be a no.**

[ ] No
[ ] Yes
[ ] Unknown

110. **ParticExtrDirection**

How can the subject's group membership development during involvement in extremism best be described?

[ ] Less time spent with the group
[ ] Stability
[ ] More time spent with the group
[ ] Abandoned the group
[ ] Unknown
[ ] Does not apply

111. **PP_Involv:** Did peer-pressures influence the radicalization process?

[ ] No
[ ] Yes
[ ] Unknown
[ ] Does not apply

112. **FTF_PreInv:** Did the individual have face-to-face ties to radicals/extremists/terrorists prior to radicalization process start?

[ ] No
[ ] Yes, radical
[ ] Yes, extremist
[ ] Yes, terrorist
[ ] Unknown
[ ] Does not apply

113. **Involve_CO:** Radicalization process characterized by cognitive opening / unfreezing?

[ ] No
[ ] Yes, cognitive opening
[ ] Yes, unfreezing
[ ] Yes, both
[ ] Unknown
[ ] Does not apply

114. **Event_CO:** Was the <u>event decision</u> influenced by cognitive opening / unfreezing?

[ ] No
[ ] Yes, cognitive opening
[ ] Yes, unfreezing
[ ] Yes, both
[ ] Unknown
[ ] Does not apply

115. **InvolveSigQ:** Was the radicalization process characterized by a clear significance quest (personal desire to matter, to have respect, to restore lost self-esteem, significance)?
[ ] No
[ ] Yes
[ ] Unknown
[ ] Does not apply

116. **EventSigQ:** Was the event decision characterized by a clear significance quest (personal desire to matter, to have respect, to restore lost self-esteem, significance)?
[ ] No
[ ] Yes
[ ] Unknown
[ ] Does not apply

117. **ExtrRoleM_PreInv:** Did the individual look up to / was he or she inspired by extremist role-models or authority figures prior to radicalization?
[ ] No
[ ] Yes
[ ] Unknown
[ ] Does not apply

118. **Recruited:** If the subject became involved in a radical, extremist or terrorist group, was he/she recruited?
[ ] No
[ ] Yes
[ ] Unknown
[ ] Does not apply

119. **BenefitsPre:** Perceived benefits of participation in extremist/terrorist group or broader extremist movement as pull factor pre-involvement?
[ ] No
[ ] Yes
[ ] Unknown

[ ] Does not apply

120. **BenefitsPost:** Perceived benefits of participation in extremist/terrorist group or broader extremist movement as involvement anchor?

[ ] No
[ ] Yes
[ ] Unknown
[ ] Does not apply

121. **RoleSoughtGiven:** Role within radical, extremist or terrorist group sought or given?

[ ] Sought
[ ] Given
[ ] Unknown
[ ] Does not apply

122. **InGroupSigQ:** Within extremist/terrorist group role adoption characterized by desire for significance within said group?

[ ] No
[ ] Yes
[ ] Unknown
[ ] Does not apply

123. **PP_RoleAdopt:** Did peer-pressures influence primary role adoption within group or broader movement?

[ ] No
[ ] Yes
[ ] Unknown
[ ] Does not apply

124. **RoleDescr:** What was the principal role that the individual occupied within the group or broader movement?

[ ] Leader
[ ] Ideologue
[ ] Recruitment
[ ] Propaganda
[ ] Finances
[ ] Logistics
[ ] Hanger-on
[ ] Violence
[ ] Viol. suprt.

[ ] Non-violent activism
[ ] Other
[ ] Unknown
[ ] Does not apply

125. **RoleDescrSecondary:** What was the secondary role that the individual occupied within the group or broader movement?

[ ] Leader
[ ] Ideologue
[ ] Recruitment
[ ] Propaganda
[ ] Finances
[ ] Logistics
[ ] Hanger-on
[ ] Violence
[ ] Viol. suprt.
[ ] Non-violent activism
[ ] Other
[ ] Unknown
[ ] Does not apply

126. **RoleDescrTertiary:** What was the tertiary role that the individual occupied within the group or broader movement?

[ ] Leader
[ ] Ideologue
[ ] Recruitment
[ ] Propaganda
[ ] Finances
[ ] Logistics
[ ] Hanger-on
[ ] Violence
[ ] Viol. suprt.
[ ] Non-violent activism
[ ] Other
[ ] Unknown
[ ] Does not apply

127. **TimeInvolved:** Length of involvement as measured from initial radicalization to arrest / end of active involvement?

[ ] *Time in months*
[ ] Unknown

[ ] Does not apply

128. **Adventure:** Notable desire for adventure / thrill of violence?
[ ] No
[ ] Yes
[ ] Unknown
[ ] Does not apply

129. **Riches:** Did a potential for material gain play a role in drawing the individual to become involved **and/or** contribute to keeping the individual involved?
[ ] No
[ ] Yes, draw
[ ] Yes, during
[ ] Yes, both
[ ] Unknown

130. **StrongNegEmoInvolv:** Strongly-experienced negative emotions prior to involvement?
[ ] No
[ ] Yes
[ ] Unknown

131. **StrongNegEmoEvent:** Strongly-experienced negative emotions prior to event decision?
[ ] No
[ ] Yes
[ ] Unknown
[ ] Does not apply

132. **EmoEscalInvolve:** Did negative emotions escalate before involvement?
[ ] No
[ ] Yes
[ ] Unknown
[ ] Does not apply

133. **EmoEscalEvent:** Did negative emotions escalate before event decision?
[ ] No
[ ] Yes
[ ] Unknown
[ ] Does not apply

134. **OverConf:** Perceived by others as overconfident or self-aggrandizing?
[ ] No

[ ] Yes
[ ] Unknown
[ ] Does not apply

135. **MortSalPre:** Notably experienced mortality salience prior to radicalization start?
[ ] No
[ ] Yes
[ ] Unknown
[ ] Does not apply

136. **MortSalPost:** Notably experienced mortality salience during involvement / radicalization?
[ ] No
[ ] Yes
[ ] Unknown
[ ] Does not apply

137. **Dehuman:** Engaged in dehumanization of opponents during involvement? (E.g. ‘unbelievers’, ‘apostates’, ‘pigs’, ‘vermin’, ‘mud people’ etc., all designations intended to place opponents in a different category of people (or even treat them as essentially inhuman), lowering psychological barriers to hurting or killing them).
[ ] No
[ ] Yes, degradation
[ ] Yes, dehumanization
[ ] Unknown
[ ] Does not apply

138. **MoralDis:** Utilized moral disengagement to diminish personal responsibility for supported, planned, or executed terrorist violence?
[ ] No
[ ] Yes
[ ] Unknown
[ ] Does not apply

139. **RevengeInvolv:** Involvement process driven by desire for revenge?
[ ] No
[ ] Yes
[ ] Unknown
[ ] Does not apply

140. **RevengeEvent:** Event decision driven by desire for revenge?
[ ] No

[ ] Yes
[ ] Unknown
[ ] Does not apply

141. **RelPractice:** Religious practice prior to involvement best described as:
[ ] Intensive
[ ] Neutral
[ ] Low
[ ] Unknown
[ ] Does not apply

142. **FearArrest:** Marked fear of arrest / injury / death / opportunity costs?
[ ] No
[ ] Yes, bodily harm or death
[ ] Yes, imprisonment
[ ] Yes, opportunity costs other
[ ] Yes, all
[ ] Unknown
[ ] Does not apply

143. **UtilityViolence:** Views on utility of violence during involvement best described as:
[ ] Preference violent means
[ ] Mixed strategy: violent and non-violent means
[ ] Preference non-violent means
[ ] Uninterested in seriously working towards realization ideological goals
[ ] Unknown

144. **RelDepPrior:** Experienced marked relative deprivation prior to radicalization?
[ ] No
[ ] Yes
[ ] Unknown

145. **RelDepDuring:** Experienced marked relative deprivation during involvement / radicalization?
[ ] No
[ ] Yes
[ ] Unknown
[ ] Does not apply

146. **ExtSupportPrior:** Presence external support systems that provide pro-social support prior to involvement (school, work, relationship with non-radicalized partner)?

[ ] No
[ ] Yes
[ ] Unknown

147. **ExtSupportDuring:** Presence external support systems that provide pro-social support during involvement (school, work, relationship with non-radicalized partner)?

[ ] No
[ ] Yes
[ ] Unknown

148. **ExtSupportDirection:** Influence of external support systems on individual is best described by:

[ ] Decrease
[ ] Consistency
[ ] Increase
[ ] Essentially ceased
[ ] Unknown
[ ] Does not apply

149. **IsolationPrior:** Degree of social isolation prior to radicalization is best described as: (Relationship = 1, having 7 or more friends/relatives = 1, school/study/work = 1, regular religious attendance = 1, sports/club/volunteer work attendance = 1. Max total = 5.[10])
Note: if one of these elements underwent noted change, count as 0,5 (e.g. had work at start of involvement but soon quit altogether = 0,5)

[ ]
[ ] Unknown

150. **IsolationDuring:** Degree of social isolation during involvement is best described as:

[ ]
[ ] Unknown

151. **IsolationDirection:** During – Prior.

[ ] More isolated
[ ] Consistency (max +/- 0,5 difference)
[ ] Less isolated
[ ] Unknown

[10] Based on adaption of: Alcaraz et al., "Social Isolation and Mortality in US Black and White Men and Women."

152. **ViewPointDiversityPrior:** Contacts with people who held different ideological views or were simply uninterested in them and in any case opposed to violence prior to radicalization?

[ ] No
[ ] Yes
[ ] Unknown

153. **ViewPointDiversityDuring:** Contacts with people who held different ideological views or were simply uninterested in them and in any case opposed to violence during radicalization?

[ ] No
[ ] Yes
[ ] Unknown

154. **ViewPointDiversityDirection:** did exposure to different viewpoints decrease, remain essentially the same or increase during involvement?

[ ] Decrease
[ ] Consistency
[ ] Increase
[ ] Essentially ceased
[ ] Unknown
[ ] Does not apply

155. **DutyOfCarePrior:** Did the individual have a duty to care, such as for children and/or elderly parents, prior to radicalization?

[ ] No
[ ] Yes
[ ] Unknown

156. **DutyOfCareDuring:** Did the individual have a duty to care, such as for children and/or elderly parents, during involvement?

[ ] No
[ ] Yes
[ ] Unknown

157. **StressorPrior:** Exposed to (recent) stressor(s) prior to radicalization process start?

[ ] No
[ ] Yes
[ ] Unknown

158. **StressorDuring:** Exposed to (recent) stressor(s) during involvement?

[ ] No
[ ] Yes

[ ] Unknown

159. **PrecipitantEvent:** If involved in (preparations for) terrorist violence, did a precipitant event play a role?

[ ] No
[ ] Yes
[ ] Unknown
[ ] Does not apply

Removed

11 June 2020

- Prosocial peers variables removed. These were too strongly based on guesstimates about the behavior / worldview of peers that subject *were likely* to interact with at places like school. The value here is essentially to gauge whether or not subject were socially exposed to people who held non-extremist views. This is already captured by the ViewPointDiversity variable, which also requires less 'looking into the heads' of people about who only guesstimates can be made (e.g. school friends).

24 August 2020

- **DistToGroup**. Very labor intensive to piece together what amounts to average travel distance to locations mentioned during radicalization involvement such as demonstrations, pubs, friends' houses, targets etc. Not accurate in any case, because someone may be most active where they live but these meetings are not recorded in detail so what happens is that the exceptions (e.g. travel to a demo 200km away) is mentioned in a source, leading to overinflated averages. Or that someone who travels to Syria or the US once from Europe suddenly has a 1000km average travel distance, which is also not a useful or accurate reflection.
